# TVFx – CoVID-19 X-Ray images classification approach using neural networks based feature thresholding technique

**DOI:** 10.1186/s12880-023-01100-8

**Published:** 2023-10-02

**Authors:** Syed Thouheed Ahmed, Syed Muzamil Basha, Muthukumaran Venkatesan, Sandeep Kumar Mathivanan, Saurav Mallik, Najah Alsubaie, Mohammed S. Alqahtani

**Affiliations:** 1 Department of Electrical Engineering, Indian Institute of Technology, Hyderabad., Hyderabad, India; 2 School of Computer Science and Engineering, REVA University, Bengaluru, India; 3 Department of Mathematics, College of Engineering and Technology, SRM Institute of Science and Technology, Kattankulathur, Tamilnadu 603203 India; 4 School of Computing Science & Engineering, Galgotias University, Greater Noida, Uttar Pradesh 203201 India; 5 Department of Environmental Health, Harvard T H Chan School of Public Health, Boston, MA 02115 USA; 6 Department of Pharmacology & Toxicology, The University of Arizona, Tucson, AZ 85721 USA; 7 Department of Computer Sciences, College of Computer and Information Sciences, Princess Nourah Bint Abdulrahman University, P.O. Box 84428, Riyadh, 11671 Saudi Arabia; 8 Radiological Sciences Department, College of Applied Medical Sciences, King Khalid University, Abha, 61421 Saudi Arabia; 9 BioImaging Unit, Space Research Centre, University of Leicester, Michael Atiyah Building, Leicester, LE1 7RH UK

**Keywords:** COVID-19, X-Ray image classification, Feature-alignment, Corona virus classification, COVID-19 detection

## Abstract

COVID-19, the global pandemic of twenty-first century, has caused major challenges and setbacks for researchers and medical infrastructure worldwide. The CoVID-19 influences on the patients respiratory system cause flooding of airways in the lungs. Multiple techniques have been proposed since the outbreak each of which is interdepended on features and larger training datasets. It is challenging scenario to consolidate larger datasets for accurate and reliable decision support. This research article proposes a chest X-Ray images classification approach based on feature thresholding in categorizing the CoVID-19 samples. The proposed approach uses the threshold value-based Feature Extraction (TVFx) technique and has been validated on 661-CoVID-19 X-Ray datasets in providing decision support for medical experts. The model has three layers of training datasets to attain a sequential pattern based on various learning features. The aligned feature-set of the proposed technique has successfully categorized CoVID-19 active samples into mild, serious, and extreme categories as per medical standards. The proposed technique has achieved an accuracy of 97.42% in categorizing and classifying given samples sets.

## Introduction

The CoVID-19 also termed as coronavirus 19 is classified as Severe Acute Respiratory Syndrome Coronavirus 2 (SAR–CoV–2) is declared a global pandemic by World Health Organization (WHO) in early 2020 as the first case reputed in Wuhan province of China in December 2019 [1]. The CoVID-19 influences human lungs from the noticeable symptoms seen in patients with lungs serge, leading to breathlessness, thus resulting in oxygen depletion causing death. As of May 2021, the global infection rate has crossed 159 Million confirmed cases and 3.32 Million reported deaths. Currently, India is on the global index with 4 lakh daily reporting cases causing a second wave of infections. The overall health infrastructure is breaking down due to a lack of expertise, facilities, resources, and vaccine. Many researchers have provided solutions in combating CoVID-19 with effective RTPCR tests, Telemedicine, and tele diagnosis tools.

The author, Ismael, A.M [2] in the research considered the X-ray images and applied deep learning approaches on X-ray image data for the detection of COVID-19. CNN models (ResNet18) are designed for feature extraction and SVM classifier with Quadratic kernel is used for classification. In the related work [3], the authors have used COVID-19 X-ray images and extracted images using single layer based and feature based techniques. The extracted features are further classified. The composite model consists of four phases: Preprocessing, deep feature extraction, feature fusion, post processing and muti-class classification. In [4], the author used weiner filtering to preprocess the input, gray level co-occurrence matrix to extract fusion based features and Artificial Neural Network is designed to perform classification. Machine Learning architecture is proposed to perform classification on covid-19 x-ray images. Histogram based gradient is used to extract features from the images and CNN model is used for classification [5].

**Fig. 1:**
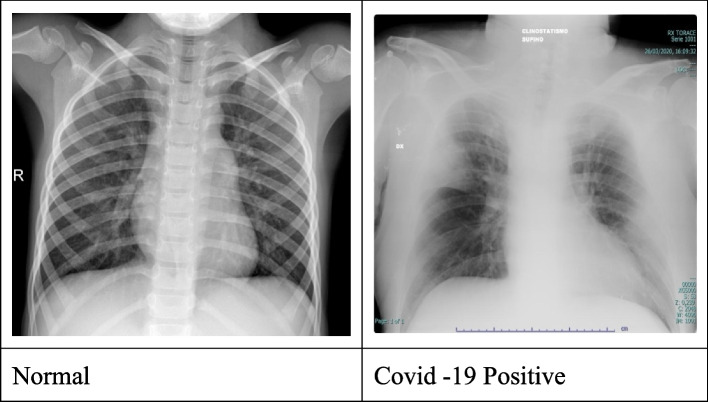
Dataset briefing of X-Rays under norma and abnormal representation

The battle against COVID-19 is much effective if detected on early grounds. Even if limited by the possibility of false negatives, chest X-Ray represents the first approach in the emergency setting [6] due to its wide availability, low cost, execution at the patient’s bed, and possibility to predict the patients’ outcome [7, 8]. Early diagnosis allows for a better patients’ management when compared to undiagnosed or late diagnosis [9]. The authors have discussed a novel approach for predicting CoVID-19 using X-Ray images using deep transfer learning. In this article, the authors have proposed an interdependent approach of feature thresholding technique based on a neural network model. The feature set, extracted aims to retrieve the most relevant features. The attribute correlation and neural networking model help the evaluation of X-rays in extracting the region of interest (ROI) from datasets. Figure 1 representing normal and abnormal (CoVID-19 Positive) X-Rays images.

The fundamental paradigm required for the validation and processing COVID-19 images via X-ray images are limited to feature-set selection and attribute based clustering using training models. In this study, we proposed a method based on thresholding approach of extracted features and clustering parameters using TVFx technique.

The purpose of this research article is to classify the COVID-19 images under minimal feature-set using thresholding approach of extracted features and clustering parameters using TVFx technique. The input dataset for the proposed technique is X-Ray images validated by the Kaggle digital library and processed under a feature distributed technique for re-alignment of datasets. The inter-connected feature set is then aligned and generated with a threshold parameter to support a rapid classification and decision support of CoVID-19.

Our main contributions in this research study are as follows:

1. Classification of COVID-19 images under minimal feature set
2. Making use of thresholding approach to extract the features
3. Clustering parameters using TVFx technique

This research paper article is organized as: literature review is discussed in section "Literature Review", methodology and design model is summarized in sections "Proposed Methodology" and "Results and Discussions", respectively. The conclusion and results are discussed on section "Limitations and Future Work" with research findings.

## Literature review

The CoVID-19 detection and early treatment is an alarming concern and thus, many researchers have proposed various solutions and methodologies in combatting this novel virus. In this regard, [10] a fast detection of CoVID-19 is proposed under an application of nCOVnet model. The dataset is processed under a dedicated channel of CNN model with a training accuracy of 93-97% with respect to lower training loss of 0.2%. The technique is based on a conclusive design of RT-PCR testing results and predication system. A similar approach of deep neural networking model is proposed by [11] using NIH chest X-rays datasets. The technique is supported by layer-wise relevance propagation (LRP) scheme in understanding the CoVID-19 datasets. The LRP approach has gained an accuracy of 100% over the testing sample set.

CovidAID is proposed by [12] under the schema of RTPCR testing and validation. This is an AI supported deep learning model in predicting patients testing and ratio of positivity rate evaluation. The model has claimed 90% accuracy in detection and modeling of CoVID-19 detection and prediction under 100% sensitivity under recalling of CoVID-19 inflections. The focus is made on pulmonary disease [13] and coronavirus detection system. The system is aimed to retrieve an accuracy of 99% on a delay rate of 2.5 sec under provided datasets. The approach is streamlined by three layer validation and modeling schema, with each layer modeling the phenomenon of predictive analysis and mapping. The inter-mapping of CXR’s normal is discussed in [14] with a accuracy record of 99.1% in the given dataset model. The technique is supported by empirical evaluation models in computing weighted average of best-performing models in classification. The ROI based extraction discusses a reliable approach of feature mapping and decision supporting.

The process of detection using deep learning is processed under an automation [15] process using the predictive results of RT-PCR. The process utilizes the weight-bounded validation of datasets and features under well-trained networks of small datasets. The proposed technique has a limited scope for validation and evaluation. The overall system proposed has a higher order of accuracy and performance estimation [16]. The AI and Machine learning tools aim the prediction model a much reliable efficacy and performance estimation. The artificial intelligence model assures the forecasting and prediction of CoVID-19 under a medical paradigms and infrastructure such as MooM datasets [17] models under telemedicine environment. The further evaluation is processed using IoT framework of Depth wise separable CNN models termed as DWS-CNN for diagnosis and classification of CoVID-19.

The process of classifying COVID-19 under given X-ray images dataset, the process of independent techniques are evaluated as shown in Table 1. The process includes a variation of datasets with respect to training features. The cumulative outcome of accuracy is dependent on sensitivity, precision and F1-score. Research gaps identified based on the literature review are with reference to a limited domain of training datasets. The training datasets acquired from the existing repositories are untimely updated. Henceforth, computing the decision support based on repositories (dynamic) is a complicated system. Thus, a research gap is identified to resolve the dependencies of raining datasets bind to a dedicated repository by introducing the proposed threshold based classification approach for reliable decision support [18, 19].

**Table 1:** Survey on COVID-19 X-ray images dataset

Ref. no	Datasets Size	Accuracy	Sensitivity	Precision	F1 Score
[15]	50	90	100	100	91
[16]	100	98	96	100	98
[17]	13,975	96.23	100	-	100
[20]	381	95.33	95.33	-	95.34
[21]	5863	99.00	98.97	98.97	98.97
[22]	502	88.10	96.40	-	84.40

The literature details on the multi-dimensional dataset processing [20] with reference to the interdependency parameter evaluation and customization. The datasets of CoVID-19 is considered via the replicative approach of MOTIF patterns to extract the threshold values. The relevance of optimization in processing the COVID-19 datasets is discussed and validated by [21] using a Bayesian optimization approach. The technique assures a reliable decision support using CNN architecture. The approach parallel to the process is the scope of identifying comorbidities related illness such as [22] with a cardio related diseases prediction and validation. The comorbidities of CoVID-19 is further discussed and surveyed in [23]. The survey observations note that, the dataset class of CoVID-19 is relatively larger and needs to be customized in the format of multiple datasets. The further evaluation process on CoVID-19 at universal level is larger and hence a sophisticated approach of customizing datasets via training and archiving is required. The proposed threshold based validation approach is an primary solution for larger dataset optimization and processing.

## Proposed methodology

**Fig. 2:**
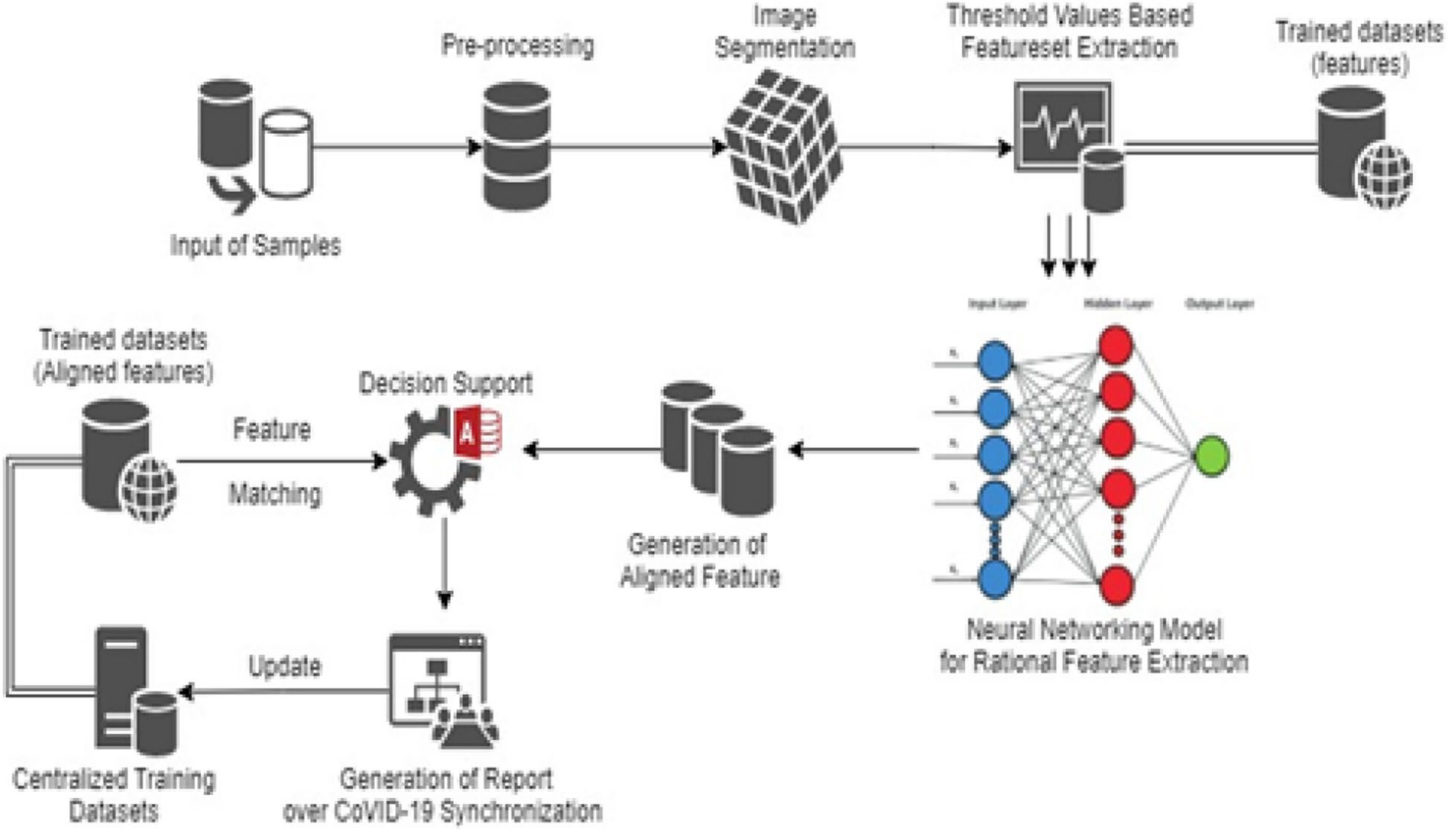
Architecture of proposed TVFx technique

**Fig. 3:**
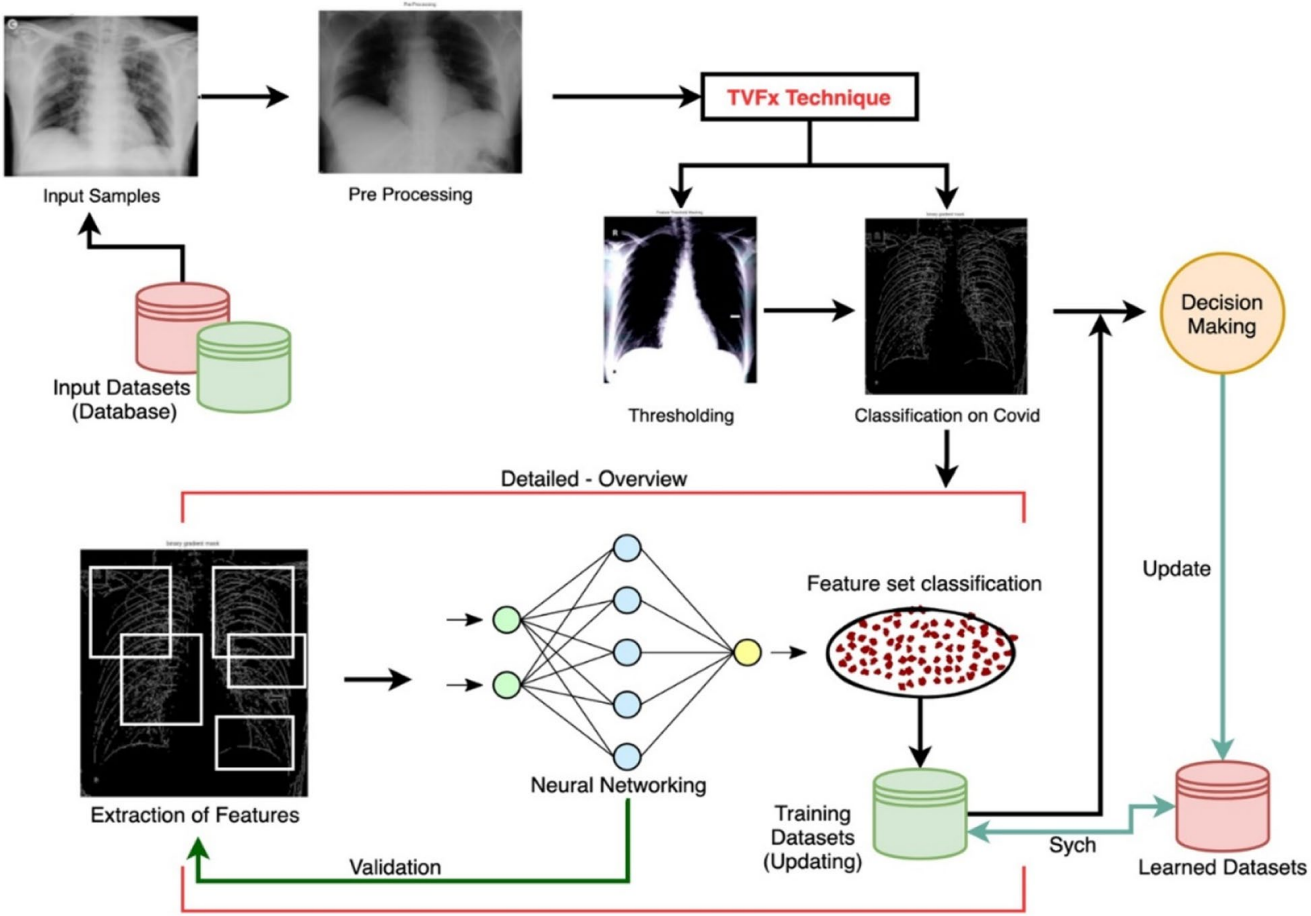
Block-diagram of proposed TVFx technique

The proposed technique is supported by an aligned feature set-based thresholding framework for classification and decision support of CoVID-19. The approach is supported and coordinated by pre-processing of X-Ray images under noise removal and image segmentation approach to categorize the labels in processing datasets as shown in Figs. 2 and 3 respectively. The proposed model assures the processing of datasets (CoVID-19 X-Rays) with an objective of threshold values-based feature extraction. The image segmentation is processed with feature clustering and categorizing the datasets using the threshold values-based feature extraction (TVFx) technique. The TVFx technique updates the trained datasets to assure a reliability factor in decision support for initial processing. The TVFx approach of the feature set is discussed in Section "Results and Discussions" with an intermediate assessment factor of feature alignment using a neural networking model. The aligned vector of features are internally dependent on the extracted attribute of Covid related X-Rays compared to Non-Covid datasets.

The decision support is then processed and aligned with a dual-mode of information matching and validation with reference to trained threshold value matching for an unambiguous decision support in classifying the dataset sample as Covid positive. The summarization is further extended towards report generation and validation. The proposed TVFx technique assures the decision support to be validated under a dual inter-dependent correlation of attributes.

The decision support vector in the proposed TVFx technique is aided under the influence of a dedicated neural networking ecosystem. The support of features and attributes correlation is summarized into the intermediate trained datasets named as “aligned features”. These intermediate datasets are processed and combined with trained datasets towards supportive decision-making. The TVFx technique is thus updated to centralized training datasets for providing a reliable decision trained and self-learned approach.

### Thersholding value based feature (Tvfx) technique

Under the proposed technique of “Feature Thresholding” for CoVID-19 X-Ray images classification, the predictive model uses a dedicated Neural networking model and an independent feature set evaluation approach. Consider the input set (S) of set of CoVID samples as $S = \left\{ {S_{1} ,S_{2} ,S_{3} ...S_{n} } \right\}$ the values associated with each internal segment attains a higher order of attribute (A) correlation as $\left( {\forall A_{n} \subset S_{i} \cup S_{i} \in S} \right)$ such that, each functional value of Ai in Si is a resultant function attribute set. Typically, the attributes are then categorized into features (F).

Each of feature set (F) is functionally associated with a series of interconnected attributes set i.e. $\left( {\forall F \subseteq A} \right)$ in an outlook ecosystem. Typically, the fundamental features are termed as interconnected attributes pairs. The pair functions can be represented as $\left( {\left( {F_{S} } \right)_{i} \subseteq A_{i} \emptyset \forall A_{1,2,3...n} \in S} \right)$ these attributes feature set is then co-associated with existing functional values. The study demonstrated a continuous array of CoVID-19 feature sets that constitute a continuous array of ***S***_***C***_ such that, represented in Eq. 1 where $\Delta T_{x}$ is a threshold value of feature learned set as to be discussed in Algorithm 1.

1 $${{\varvec{S}}}_{{\varvec{C}}}=\sum\nolimits_{{\varvec{i}}={\varvec{o}}}^{\boldsymbol{\infty }}(\boldsymbol{\Delta }{{\varvec{T}}}_{{\varvec{x}}}\sum\nolimits_{{\varvec{j}}}^{{\varvec{n}}}\frac{{\varvec{\delta}}{({{\varvec{F}}}_{{\varvec{S}}})}_{{\varvec{j}}}}{{\varvec{\delta}}{\varvec{t}}}\times \frac{{\varvec{\delta}}{({{\varvec{A}}}_{{\varvec{i}}})}_{{\varvec{j}}}}{{\varvec{\delta}}{\varvec{t}}})$$

### CoVID-19 image processing

With functional parameters of CoVID-19 focused datasets (SC) the resultant input data is further segmented into smaller order of attributes correlation, the segmented attributes with respect to incoming signals is related as Eqs. 2 and 3 respectively

2 $$S=[\frac{{\mathrm{lim}}_{n\to \infty }(\frac{\delta ({S}_{C})}{\delta t})\Delta {T}_{x}}{\frac{\delta ({A}_{i})}{\delta t}}]$$

3 $$S=[\frac{{\mathrm{lim}}_{n\to \infty }(\frac{\delta ({S}_{C})}{\delta t})\Delta {T}_{x}}{\frac{\delta ({A}_{i})}{\delta t}}]$$

With inclusion of iterative function, the functional attributes of $\delta \left( {S_{C} } \right)$ and $\delta \left( {A_{i} } \right)$ is represented as Eq. 4

4 $${{\varvec{S}}}_{{\varvec{L}}}={\mathbf{l}\mathbf{i}\mathbf{m}}_{{\varvec{n}}\to \boldsymbol{\infty }}[\sum\nolimits_ {{\varvec{j}}=0}^{\boldsymbol{\infty }}[\frac{{\varvec{\delta}}{({{\varvec{S}}}_{{\varvec{C}}})}_{{\varvec{i}}}\times{\varvec{\delta}}{({{\varvec{A}}}_{{\varvec{i}}})}_{{\varvec{j}}}}{{\varvec{\delta}}({\varvec{t}})}]]\times \boldsymbol{\Delta }{{\varvec{T}}}_{{\varvec{x}}}$$

The system is functionally extracted in threshold feature set. The feature set of each segment is represented in $\delta \left( {S_{i} } \right)$ and feature set (F), the retrieval of inter-collateral features extraction is as Eqs. 5 and 6.

5 $${\boldsymbol{F}}_{\boldsymbol{s}}=\frac{\boldsymbol{2\pi}}{\boldsymbol{\Delta{T}_x}}\left(\int^{\infty}_{-\infty}\frac{{\boldsymbol{\delta}}{\left({\boldsymbol{S}}_{\boldsymbol{C}}\right)}}{{\boldsymbol\delta}{\boldsymbol{t}}}\right)\begin{array}{l}\boldsymbol{n}\\\boldsymbol{0}\end{array}$$

The process is assigned with an interconnected set of features set (FS), extracted with reference to $\delta \left( {S_{C} } \right)$. These segments retrieve attributes and a threshold value of segments is computed.

6 $${\varvec{T}}={\mathbf{l}\mathbf{i}\mathbf{m}}_{{\varvec{n}}\to \boldsymbol{\infty }}[{\sum\nolimits_ {{\varvec{i}}=0}^{{\varvec{n}}}\sum\nolimits_ {{\varvec{j}}={\varvec{i}}+1}^{{\varvec{n}}-1}[\frac{{\varvec{\delta}}{({{\varvec{S}}}_{{\varvec{C}}})}_{{\varvec{i}}}+{\varvec{\delta}}{({{\varvec{F}}}_{{\varvec{S}}})}_{{\varvec{j}}}}{{\varvec{\delta}}{\varvec{t}}}]}^{-2}\times \boldsymbol{\Delta }{{\varvec{T}}}_{{\varvec{x}}}]$$

where, (T) is the threshold value of overall segments $\delta \left( {S_{C} } \right)$ with reference to feature set attributes, the overall attributes threshold is assigned and validated to $\left( {\Delta T_{x} } \right)$ with values assigned to each feature set. The features are then proposed with a dedicated neural networking channel for “Rational” feature extraction as shown in Eqs. 7 and 8 respectively.

7 $$\begin{array}{cc}\mathbf{T}=\mathbf{l}\mathbf{i}\mathbf{m}_{(\mathbf{n}\to \mathbf{\infty })}& ={\mathbf{l}\mathbf{i}\mathbf{m}}_{{\varvec{n}}\to \boldsymbol{\infty }}[{\sum_{{\varvec{i}}=0}^{{\varvec{n}}}\sum_{{\varvec{j}}={\varvec{i}}+1}^{{\varvec{n}}-1}[\frac{{\varvec{\delta}}{({{\varvec{S}}}_{{\varvec{C}}})}_{{\varvec{i}}}+{\varvec{\delta}}{({{\varvec{F}}}_{{\varvec{S}}})}_{{\varvec{j}}}}{{\varvec{\delta}}{\varvec{t}}}]}^{-2}\end{array}$$

Now, the rational feature are extracted and evaluated with generation of aligned features, typically, the aligned feature are the intersecting features of main (raw) datasets features with respect to feature set (F) and resultant aligned features (rational) as demonstrated in Eq. 8, the resultant feature are assured with dependency matrix set of feature resultant to CoVID-19 classification.

8 $${\varvec{T}}={\mathbf{l}\mathbf{i}\mathbf{m}}_{{\varvec{n}}\to \boldsymbol{\infty }}[{\sum\nolimits_ {{\varvec{i}}=0}^{{\varvec{n}}}\sum\nolimits_ {{\varvec{j}}={\varvec{i}}+1}^{{\varvec{n}}-1}[\frac{{\varvec{\delta}}{({{\varvec{S}}}_{{\varvec{C}}})}_{{\varvec{i}}}+{\varvec{\delta}}{({{\varvec{F}}}_{{\varvec{S}}})}_{{\varvec{j}}}}{{\varvec{\delta}}{\varvec{t}}}]}^{-2}\times \boldsymbol{\Delta }{{\varvec{T}}}_{{\varvec{x}}}]$$

Thus, the study technique suggest the functional values are streamline into an overall feature set “Aligned” on. Hence the values are further expanded with evaluation in decision support as discussed in Table 1 under the Neural Networking threshold folds sequence evaluation.

### Decision support matrix

The feature set (aligned) i.e. $\Gamma$ is a functional values of given features dependencies with respect to the scale of evaluation. The stream function for decision support is as follows in Eq. 9

9 $${{\varvec{D}}}_{{\varvec{S}}}=\frac{{\varvec{P}}({\varvec{T}}{\varvec{N}})+{\varvec{P}}({\varvec{N}})}{{\varvec{P}}({\varvec{T}}{\varvec{N}}-1)}$$

Thus, the function decision support is classified the given CoVID-19 samples into mild, medium and extreme categories with reference to given datasets. These categories are functional in nature and thus validated under medical observation. The datasets are centric towards the consideration of X-Ray datasets for providing a rational comparison.

**Figure:**
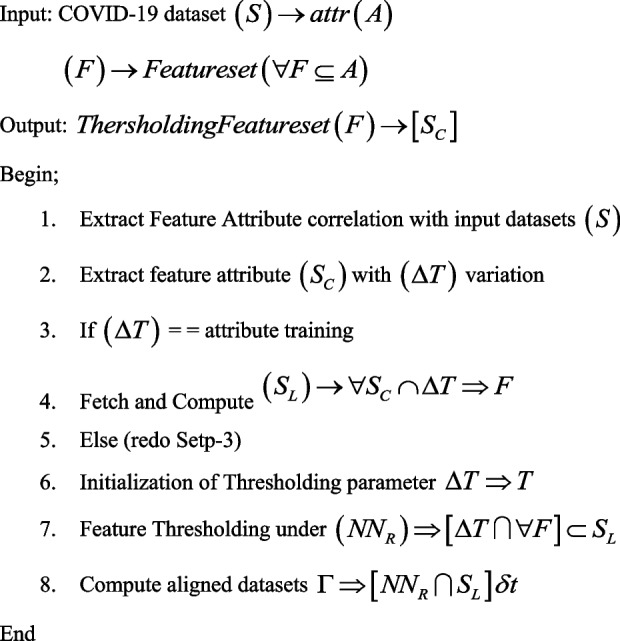
**Algorithm 1.** Feature Thersholding (TVFx) Technique

## Results and discussions

The proposed technique features (extracted) and features (aligned) are inter-computed using a dedicated channel of neural networking model. The system is supported via a MATLAB 2015R based image processing tool kit. The model is simulated on a regional matrix of 155 datasets of X-Ray images with a comparative outcome observed in Table 2 with supporting matrix of processing data samples in Table 4. These datasets are expressed on the classification of normal and abnormal COVID datasets into a stream of trained datasets. The CoVID-19 dataset is considered from Kaggle and Github directory, the validating threshold values of CoVID-19 dataset is extracted from Cancer of Image Repository (CIA). The 147 datasets are analysis and shortlisted based on the feature set and contribution dimension.

**Table 2:** Classification and feature evaluation based on thersholding of nn fold sequences

Fold Sequence	Sensitivity	Specification	Precision	Threshold Recall	Range Accuracy
Fold 1	92.23	91.82	93.2	95.82	96.752
Fold 2	95.54	90.38	89.2	93.19	96.23
Fold 3	90.11	89.37	82.12	89.02	96.23
Fold 4	90.94	90.01	87.23	89.45	97.24
Fold 5	91.23	92.47	90.38	90.34	98.34
Fold 6	97.23	94.63	94.21	96.93	98.23
Fold Avg	92.88	91.44667	89.39	92.45833	**97.42**

**Fig. 4:**
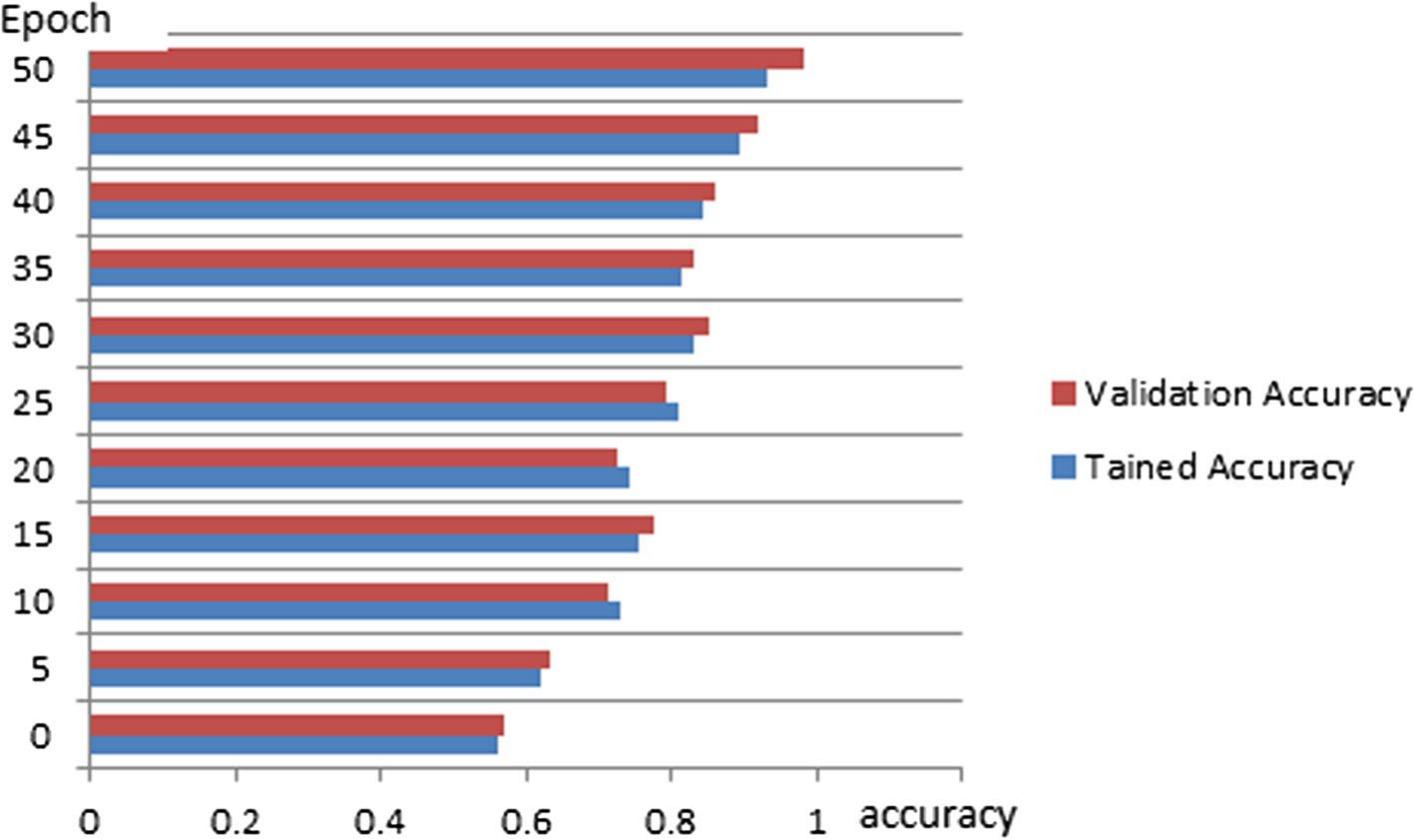
Comparative graph on validation and trained accuracy

**Fig. 5:**
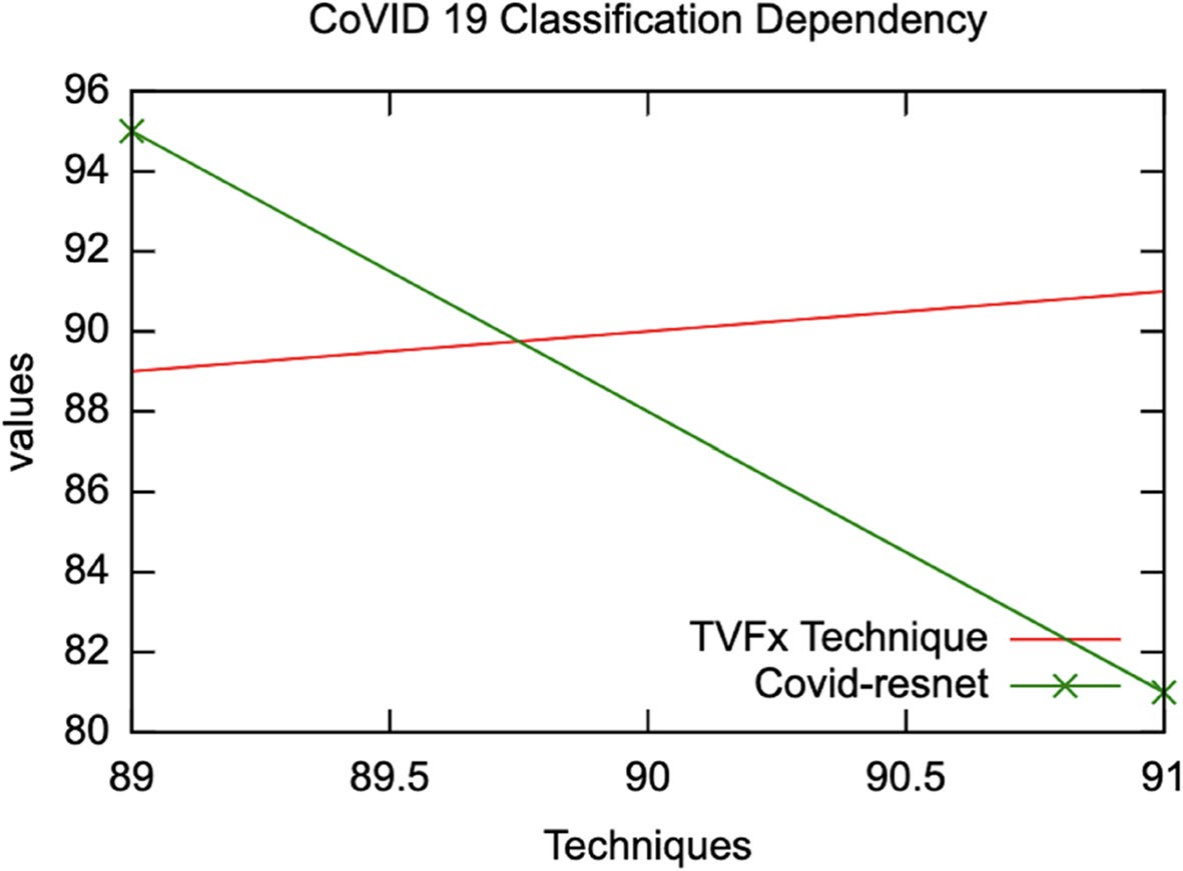
Dependency evaluation of TVFx technique

**Fig. 6:**
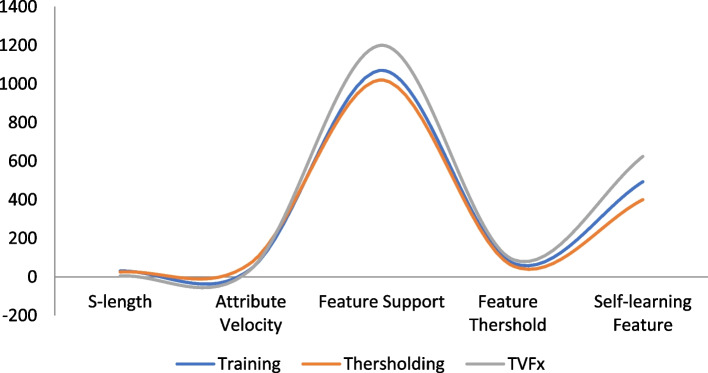
Comparative feature observation

The proposed schema has evaluated the input samples via a series of processing and implementation as shown in Table 3. The comparative analysis of the proposed schema is studied and reported in Table 4 with proposed technique achieving 97.17% in performance accuracy under correlated parameters of self-leaning features. The Table 3 dataset samples are preprocessed and demonstrated in an order of extracting ROI segment from the processed and aligned datasets generated via Neural Networking ecosystem. A detailed representation of comparative graphs on validation and trained accuracy is shown in Fig. 4 and dependency valuation in Fig. 5. The proposed TVFx technique statics and observation parameters are discussed in Fig. 6 and Tables 5 and 6 with respect to threshold and training validation of information.

**Table 3:**
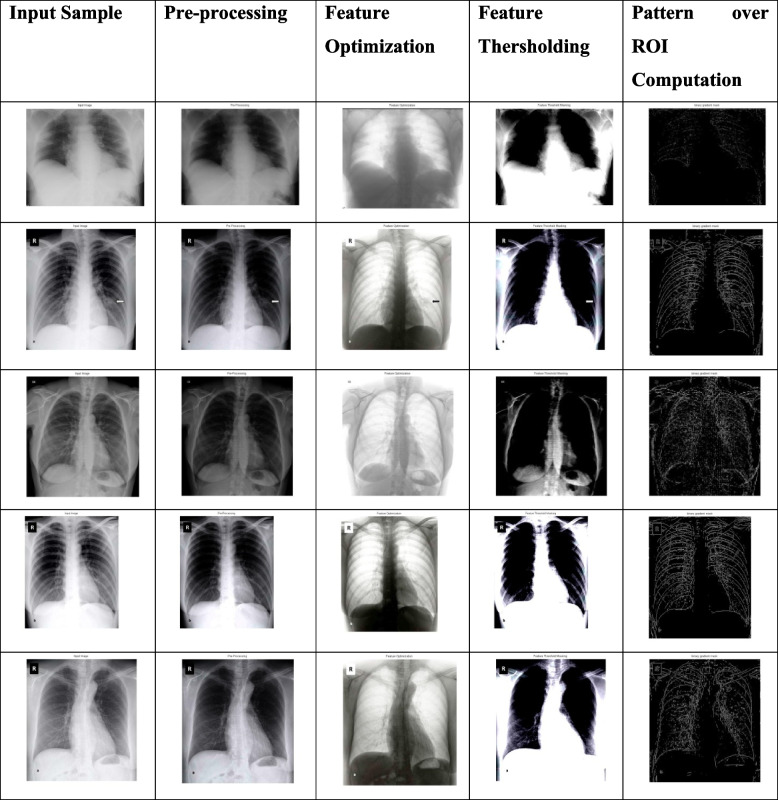
Processing of CoVID-19 positive datasets under proposed schema

**Table 4:** Performance computation (Domain Categorization)

Techniques	Accuracy	Range	Mild	Server	Extreme	Performance
Structural	88.29	0–12	√			91.28
Sequential Processing	81.24	0–12	√	√		90.17
Deep Learning	91.49	0–15	√	√	√	96.23
TVFx Proposed technique	97.42	0–15	√	√	√	**97.17**

**Table 5:** Performance computation (Independent Techniques)

Techniques	Datasets Size	Training Size	F1 Score	Accuracy
Covidx-net [24]	50	10	91	90
DNN [25]	100	40	98	98
Covid-resnet [26]	13,975	282	100	96.23
Deep Learning Model [27]	13,800	152	91.22	93.90
CXR features set [28]	502	126	84.40	88.10
**TVFx Proposed technique**	**661**	**274**	**95.82**	**97.42**

**Table 6:** Computation of performance matrix of the proposed TVFx technique

Training dataset sample size	Validating dataset sample size	Epoch	F1 Sore	Precision	Recall
178	483	10	93.82	88.82	89.42
178	483	20	93.92	89.04	90.43
274	387	10	94.76	89.52	90.07
274	387	20	95.82	90.43	90.88

## Limitations and future work

The proposed system has recorded a performance efficiency of 97.17% over 155 COVID X-Ray image datasets with reference to trained accuracy and validated accuracy as discussed in Results and discussion section, the validated accuracy has achieved a higher order of performance compared to trained datasets. The technique has primarily supported the prediction and classification of CoVID-19 disease under regular X-Ray samples. The prediction ration of implementation using proposed TVFx technique with reference to the value projection of features and attributes velocity of correlation.

## Conclusion

The proposed technique has successfully classified and extracted CoVID-19-positive samples and categorized them into mild, medium, and serious bandwidth. The proposed technique, i.e. feature thresholding approach on interconnecting and feature evaluation has demonstrated a higher order of prediction accuracy. The proposed system has recorded a performance efficiency of 97.17% over 155 COVID X-Ray image datasets with reference to trained accuracy and validated accuracy, the validated accuracy has achieved a higher order of performance compared to trained datasets. The technique has primarily supported the prediction and classification of CoVID-19 disease under regular X-Ray samples. The prediction ratio of implementation using the proposed TVFx technique with reference to the value projection of features and attributes velocity of correlation. The TVFx technique is subjected to a self-learning framework for training and validating COVID-19 datasets in the near future.

## Data Availability

The datasets used during the current study are available from the corresponding author on reasonable request.
